# Patient reported long-term outcome after endovascular therapy of indirect dural carotid cavernous fistulas

**DOI:** 10.1371/journal.pone.0231261

**Published:** 2020-04-10

**Authors:** Lorenz Ertl, Hartmut Brückmann, Maximilian Patzig, Franziska Dorn, Gunther Fesl

**Affiliations:** 1 Institute of Neuroradiology, University Hospital, LMU Munich, Munich, Germany; 2 Radiologie Augsburg-Friedberg ÜBAG, Augsburg, Germany; Universitatsklinikum Freiburg, GERMANY

## Abstract

**Purpose:**

Patient-reported long-term follow-up after endovascular treatment of indirect carotid cavernous fistulas is important, but rarely addressed in literature. We report on this issue with a special focus on the patient’s view and its impact on the indication evaluation process.

**Methods:**

We retrospectively reviewed the records of all patients (n = 33) with a minimum follow-up interval of at least 36 and up to 166 months after endovascular treatment of an indirect carotid cavernous fistula (Barrow B-D) at our institution (treated from 01/2003 to 06/2015). We determined treatment details including primary therapy success and complication rate and quote the patient’s subjective perception of the long-term treatment success using a standardized interview form.

**Results:**

As a primary result the fistula was completely occluded in 25/33 cases (76%), while a downgrading was achieved in 8/33 (24%) of the cases. Secondary occlusion was observed in three out of eight patients (38%). In the long-term interview (response rate: 91%, median follow-up interval: 114 months) 87% of the patients reported high satisfaction with the long-term therapy result. Endovascular treatment achieved a sustainable relief from all eye-related symptoms in 89% and from pulsatile tinnitus in 57% of the cases.

**Conclusions:**

In addition to good results in terms of angiographic and clinical cure, endovascular treatment is also perceived as beneficial by most of the patients. This is another important argument in favor of an endovascular treatment.

## Introduction

Carotid cavernous sinus fistulas (CCF) are abnormal arteriovenous shunt connections between the carotid circulation and the cavernous sinus. They are commonly classified into four types (A-D) according to Barrow [1]. Whereas a Type A CCF is a direct arteriovenous shunt, the Types B-D present as an indirect communication between dural branches of the carotid arteries through the wall of the cavernous sinus (idCCF). The prognosis and endovascular treatment methods of both entities significantly differ [2–5].

Symptoms of an idCCF include paresis of some combinations of the cranial nerves III, IV and VI, periorbital or retro-orbital pain, and conjunctival chemosis [6]. Exophthalmos, elevated intraocular pressure (IOP), and a decrease of the visual acuity can be observed as well. An idCCF is associated with serious morbidity, threatening blindness, stroke, and cerebral hemorrhage in up to 30% to 40% of lesions associated with retrograde venous drainage to the cerebral veins (CVD) [7,8].

Endovascular techniques as a treatment option for idCCF have emerged over the past years and there are some publications on the complication and success rates of this method [8–10]. But whereas indication to treat is indisputable in fistulas associated with cortical venous drainage (CVD), neurologic deficits, intradural hemorrhage, venous thrombosis, or altered mental status [8] any other clinical presentation of an idCCF only offers a relative treatment indication. Some authors even recommend to manage these lesions expectantly awaiting potential spontaneous closure of the fistula [4,8,11,12].

In these constellations and especially when consulting elderly patients whether to treat or not, it is not only about good angiographic results–rather an intervention is only justified if a sustainable subjective improvement of the patient's condition is to be expected.

This aspect of patient reported outcome is little addressed in the interventional radiologic literature. In this work we report on this issue by evaluating the subjective satisfaction of 33 patients with a minimum of 36 months follow-up after endovascular therapy of an idCCF. We aim to add another patient-centered perspective to the indication finding process.

## Material and methods

### Study population

We reviewed the PACS, the electronic database and the paper based medical documentation of our hospital to identify all patients who received endovascular treatment of a CCF Barrow Type B-D (idCCF) at the neuroradiology department of our institution from 01/2003 to 06/2018. From these we included all cases with a minimum of 36 months follow-up period (treated from 01/2003 to 06/2015).

### Evaluation criteria

All patient records were reviewed to determine the clinical symptoms at the time of initial presentation and during the follow-up period. The imaging material was re-analyzed in detail by two experienced neuroradiologists (LE and GF, 8 and 16 years of experience respectively). The vascular treatment approach, materials used, and any complications related to the intervention with regard to vessel dissections, thromboembolic events, hemorrhages, infarctions, lasting disabilities and death were recorded [10].

To determine the primary success rate, the final angiographic result of the last endovascular session was assigned to one of four categories: (1) complete occlusion (CO), (2) downgrading (D), (3) unchanged (U), (4) upgrading (UP, = increased AV-shunt volume).

Downgrading (D) was defined as a reduction of the AV-shunt volume in digital subtraction angiography compared to the state prior to the intervention. Residual AV-shunt volume had strictly to be confined to the sinus and must not have involved the cortical veins (= no evidence for cortical venous drainage, CVD) [10].

To evaluate the long-term success and patient reported outcome a self-designed standardized questionnaire was sent to all patients. We asked them about their clinical symptoms on admission, at the time of discharge and at the time of the interview. Finally, a summary statement on the development of their fistula-related symptoms over time by choosing one out of three categories (“better”, “equal”, “worse”) was requested and a free-comment text field was provided (S1 File, [10]).

### Interventions

All endovascular interventions were performed by trained neuroradiologists on a biplane neuroangiography suite (Neurostar Top or Axiom Artis zee biplane, both Siemens AG, Healthcare Sector, Erlangen, Germany) [10].

### Data collection and statistical analysis

All data was collected in a custom-designed database using standard software (Access 2010; Microsoft, Redmond, WA, USA). All statistical analysis was performed using MS Excel 365 (Microsoft, Redmond, WA, USA).

### Ethics statement

The ethics committee of our institution approved this study (ID: 204–15, Ethikkommission bei der LMU München). Written informed consent to participate in this study was provided by all patients.

The authors LE, MP and GF are employed by a commercial company ‘Radiologie Augsburg-Friedberg ÜBAG’–an owner-managed provider of diagnostic radiological services in the German healthcare system. The funder provided support in the form of salaries for authors LE, MP and GF, but did not have any additional role in the study design, data collection and analysis, decision to publish, or preparation of the manuscript. The specific roles of these authors are articulated in the ‘author contributions’ section. The authors state, that there are no competing interests between their employment in the ‘Radiologie Augsburg-Friedberg ÜBAG’ and this publication. Additionally this does not alter our adherence to PLOS ONE policies on sharing data and materials.

## Results

### Study population

We retrospectively identified and analyzed 33 patients (23 female / 10 male) with a minimum of 36 months follow-up after endovascular treatment of an idCCF at our institution (treated from 01/2003 to 06/2015). Median age at the time of treatment was 66 years (range: 20-84y). Two patients already had undergone unsuccessful treatment attempts in other hospitals (1x insufficient coil embolization, 1x particle embolization followed by iatrogenic ischemic stroke) (Table 1).

**Table 1:** Patient data & treatment details.

Pat ID	Pat Sex	Date treatment	Patient age (treatment)	Symptoms prior to treatment as documented in the medical records	Treatment sessions	Catheters placed	Approach used for treament	Material	Primary result	Complication
#1	F	04/2003	66	Exophthalmos, chemosis, retroorbital pain, pulsatile tinnitus	1	TA/TV	TV	COILS / HISTO	CO	None
#2	F	05/2003	65	Exophthalmos, chemosis, retroorbital pain, pulsatile tinnitus	1	TA/TV	TA/TV	COILS / HISTO	CO	None
#3	F	06/2003	63	Exopthalmos, diminished visual acuity	1	TA/TV	TV	COILS / HISTO	CO	None
#4	M	09/2004	53	Exophthalmos, chemosis, diminished visual acuity, elevated IOP	1	TA/TV	TV	COILS / HISTO	D	None
#5	M	09/2004	73	Exophthalmos, chemosis, diminished visual acuity	1	TA/TV	TV	COILS / HISTO	D	None
#6	M	06/2005	54	Exophthalmos, chemosis, diminished visual acuity, abducens nerve palsy, elevated IOP	1	TA/TV	TV	COILS / HISTO	D	None
#7	F	12/2005	63	Exophthalmos, chemosis, diminished visual acuity	1	TA/TV	TV	COILS / HISTO	CO	None
#8	M	01/2006	54	Exophthalmos, chemosis, diminished visual acuity, abducens and trochlaris nerve palsy, diplopia	1	TA/TV	TV	COILS / GLUEBRAN	CO	None
#9	F	03/2006	79	Exophthalmos, chemosis, diminished visual acuity, pulsatile tinnitus	1	TA/TV	TV	COILS / GLUEBRAN	CO	None
#10	M	07/2006	20	Exophthalmos, chemosis, elevated IOP	1	TA/TV	TV	COILS / GLUEBRAN / ONYX	CO	None
#11	F	08/2006	49	Exophthalmos, chemosis, diminished visual acuity, pulsatile tinnitus	1	TA/TV	TV	COILS	CO	None
#12	F	06/2007	59	Headache, diminished visual acuity, elevated IOP, abducens nerve palsy, CVD	1	TA/TV	TV	COILS	CO	None
#13	F	06/2007	76	Exophthalmos, chemosis	1	TA	TA	GLUEBRAN	D	None
#14	F	10/2007	83	Diminished visual acuity, pulsatile tinnitus, extensive AV-shunt with right hemispheric steal and fluctuating neurologic deficits	1	TA	TA	ONYX / BALLOON	CO	None
#15	F	08/2008	66	Exophthalmos, chemosis, pulsatile tinnitus	1	TA/TV	TV	COILS	CO	None
#16	M	09/2008	48	Exophthalmos, chemosis, diminished visual acuity	2	TA/TV	TV	COILS / ONYX	CO	None
#17	F	01/2009	69	Exophthalmos, chemosis, pulsatile tinnitus	1	TA/TV	TV	COILS	CO	None
#18	F	05/2010	79	Exophthalmos, chemosis	1	TA	TA	ONYX	SO	None
#19	F	07/2010	75	Exophthalmos, chemosis, diminished visual acuity, elevated IOP	1	TA	TA	ONYX	SO	None
#20	F	03/2011	73	Exophthalmos, chemosis, diplopia, pulsatile tinnitus	1	TA/TV	TV	COILS	SO	None
#21	M	03/2012	66	Pulsatile tinnitus	1	TA/TV	TV	COILS	D	Permanent unilateral abducens paresis
#22	F	11/2012	78	Exophthalmos, chemosis, diminished visual acuity, elevated IOP, CVD	1	TA/TV	TV	COILS	CO	None
#23	M	03/2013	49	Exophthalmos, chemosis, diminished visual acuity, pulsatile tinnitus	1	TA/TV	TV	COILS	CO	None
#24	F	06/2013	81	Exophthalmos, chemosis, diminished visual acuity, diplopia, CVD	1	TA/TV	TV	COILS	CO	None
#25	F	09/2013	84	Exophthalmos, chemosis, diminished visual acuity, ptosis, elevated IOP	1	TA/TV	TV	COILS	CO	None
#26	M	09/2013	61	Exophthalmos, chemosis, diminished visual acuity, elevated IOP, CVD, partial thrombosis of the cavernous sinus	1	TA/TV	TV	COILS	CO	None
#27	F	02/2014	83	Exophthalmos, chemosis, diminished visual acuity	1	TA/TV	TV	COILS	CO	None
#28	F	02/2014	82	Exophthalmos, chemosis, diminished visual acuity, pulsatile tinnitus	1	TA/TV	TV	COILS	CO	None
#29	F	04/2014	72	Diminished visual acuity, oculomotoric nerve palsy, ptosis	1	TA/TV	TV	COILS	CO	None
#30	F	07/2014	71	Exophthalmos, chemosis, headache, abducens nerve palsy, CVD	1	TA/TV	TV	COILS	CO	None
#31	F	07/2014	61	Exophthalmos, chemosis, diminished visual acuity, pulsatile tinnitus	1	TA/TV	TV	COILS	CO	None
#32	M	11/2014	50	Exophthalmos, chemosis, diminished visual acuity, diplopia, elevated IOP, pulsatile tinnitus, CVD	1	TA/TV	TV	COILS	CO	None
#33	F	06/2015	55	Exophthalmos, chemosis, CVD, pulsatile tinnitus	2	TA/TV	TA/TV	COILS / GLUEBRAN	CO	None

Abbreviations: TA = Transarterial, TV = Transvenous, HISTO = Histoacryl, IOP = intraocular pressure, CVD = cortical venous drainage, AV = arteriovenous, CO = complete occlusion, D = Downgrade, SO = Secondary Occlusion

### Initial symptoms & treatment details

Chemosis, exophthalmos, retroorbital pain and / or ophthalmoplegia was documented in 31/33 patients (94%) prior to the intervention. In 22/33 cases (67%) the visual acuity was diminished. 9/33 patients presented with an elevated IOP (27%). In 14 out of 33 cases (42%) a pulsatile tinnitus was documented. Two patients (6%) presented with intracranial hemorrhage due to cortical venous drainage, of which one suffered from an epileptic state upon admission. Cortical venous drainage was angiographically present in 7/33 cases (21%) (Table 1).

31/33 patients were treated in a single endovascular procedure (94%), while in 2 cases (6%) two treatment sessions were performed (Table 1).

29/33 patients (88%) had a venous and arterial access, while 4/33 patients (12%) only had an arterial access. The approach finally used for the embolization was the transarterial catheter (TA) in 4/33 patients (12%), the transvenous catheter (TV) in 27/33 patients (82%), while in two patients (6%) both catheters (TA/TV) were used (Table 1).

Material used included a combination of coils and gluebran (3/33, 9%), a combination of coils, gluebran and Onyx (1/33, 3%), a combination of coils and histoacryl (7/33, 21%) and a combination of coils and onyx (1/33, 3%). One patient was treated using only gluebran (3%), two patients were treated by Onyx solely (6%) while in 17 patients only coils were deployed (52%). In one case an occlusion balloon (4 mm x 2 mm Hyperglide, Medtronic, Irvine, USA) was used in combination with Onyx (3%) (Table 1).

### Primary success rate & complication rate

Endovascular treatment led to a complete occlusion of the idCCF in 25 out of 33 cases (76%), while in 8/33 cases (24%) a downgrading was documented. Three out of those eight downgrading patients (38%) progressed to complete fistula occlusion within the first few weeks following subtotal endovascular treatment. In none of the cases did treatment lead to an unchanged or deteriorated AV-shunt and thus showed an improvement compared to the initial state in all of the cases (Table 1).

Among 33 treated patients one complication occurred (3%). This patient (pat. #21) developed a permanent unilateral abducens paresis immediately after the intervention.

### Patient reported long-term follow-up

#### Questionnaire response rate & duration

The response rate on our standardized questionnaire was 91% (30/33 patients). One patient had died in the meantime due to renal cancer. In two cases patients did not respond to our interview request for unknown cause (Table 2).

**Table 2:** Follow-up—details.

Pat ID	Exopthalmos, chemosis, diplopia, retroorbital pain			Diminished visual acuity			Pulsatile tinnitus			Overall statement
	Prior to treatment	Immediately after treatment	Current state	Prior to treatment	Immediately after treatment	Current state	Prior to treatment	Immediately after treatment	Current state	
#1	+	-	-	-	-	-	+	-	+	Better
#2	+	-	-	-	-	-	+	+	+	Better
#3	+	-	-	+	-	-	-	-	-	Better
#4	+	-	N/A	+	-	N/A	-	-	N/A	N/A
#5	+	-	-	+	-	-	-	-	-	Better
#6	+	-	-	+	-	-	-	-	-	Better
#7	+	-	-	+	-	-	-	-	-	Better
#8	+	-	-	+	-	-	-	-	-	Better
#9	+	-	-	+	-	-	+	-	-	Better
#10	+	+	+	-	-	-	-	-	-	Equal
#11	+	+	-	+	+	-	+	-	-	Better
#12	+	-	-	+	-	-	-	-	-	Better
#13	+	+	-	-	-	-	-	-	-	Better
#14	-	+	+	+	+	+	+	-	-	Worse
#15	+	-	-	-	-	-	+	-	-	Better
#16	+	-	-	+	-	-	-	-	-	Better
#17	+	-	+	-	+	+	+	-	-	Better
#18	+	+	-	-	-	-	-	-	-	Better
#19	+	-	-	+	-	-	-	-	-	Better
#20	+	-	-	-	-	-	+	+	-	Better
#21	-	-	-	-	+	+	+	+	+	Worse
#22	+	-	-	+	-	-	-	-	-	Better
#23	+	+	-	+	-	-	+	-	-	Better
#24	+	-	-	+	+	-	-	-	-	Better
#25	+	-	N/A	+	-	N/A	-	-	N/A	N/A
#26	+	-	-	+	+	+	-	-	-	Better
#27	+	-	N/A	+	-	N/A	-	-	N/A	N/A
#28	+	-	-	+	-	-	+	+	+	Better
#29	-	-	-	+	-	-	-	-	-	Better
#30	+	-	-	-	-	+	-	-	+	Worse
#31	+	-	-	+	-	-	+	-	-	Better
#32	+	+	+	+	-	-	+	-	-	Better
#33	+	-	-	-	-	-	+	+	+	Better

+ = symptomatic,— = not symptomatic

Duration of long-term follow-up (LFU) defined as the median time interval between treatment and date indicated on the questionnaire was 114 months (range: 36–166) (Tables 2 and 3, [10]).

**Table 3:** Patient reported long-term follow-up–summary.

	Symptomatic prior to treatment	Improvement immediatesustainable	Improvement Immediate temporary		Improvement Secondary sustainable	Constantly Symptomatic		Newly symptomatic Immediately after treatment	Newly symptomatic delayed during LFU
Chemosis, Exophthalmos, Retroorbital pain, Ophthalmoplegia	27/30 (90%)	20/27 (74%)	1/27 (4%)		4/27 (15%)	2/27 (7%)		1 (Pat. #14)	-
Visual acuity	19/30 (63%)	15/19 (79%)	-		2/19 (10.5%)	2/19 (10.5%)		2 (Pat. #17 + #21)	1 (Pat. #30)
Epileptic seizures	1/30 (3%)	-	-		-	1/1 (100%)		-	-
Stroke	1/30 (3%)	1/1 (100%)	-		-	-		-	-
ICH	2/30 (7%)	2/2 (100%)	-		-	-		-	-
Pulsatile tinnitus	14/30 (47%)	8/14 (57%)	1/14 (7%)		1/14 (7%)	4/14 (29%)		-	1 (Pat. #30)
Summary statement	*Better*			*Equal*			*Worse*		
	26/30 (87%)			1/30 (3%)			3/30 (10%)		

Return rate: 30/33 (91%), n = 30, Duration long-term follow-up median (range): 114 (36–166) months

Abbreviations: LFU = Long-term follow-up, ICH = intracranial hemorrhage

#### Chemosis, exophthalmos, retroorbital pain, ophthalmoplegia

27/30 patients (90%) who responded to our interview request initially presented with at least one of these symptoms. 20 of those (74%) stated an immediate and sustainable improvement. One patient (4%) reported on an immediate, however not lasting improvement, while four patients (15%) claimed a secondary sustainable recovery. 2/27 patients (7%) remained constantly symptomatic. One patient (pat # 21) was newly symptomatic due to complicative endovascular treatment (Tables 2 and 3).

#### Visual acuity

19/30 patients (63%) who responded to our interview request initially were symptomatic. 15 of those (79%) immediately and sustainably recovered, while in two patients (10.5%) the recovery was secondary during the follow-up period. 2/19 patients (10.5%) remained constantly symptomatic. Two patients (pat #17 & 21) reported on being newly symptomatic immediately after treatment while one patient (pat #30) claimed to have a secondary deterioration of his previously good visual acuity three years after successful fistula occlusion (Tables 2 and 3).

#### Epileptic seizures, stroke & intracranial hemorrhage

One patient (3%) suffered constantly from a preexisting epileptic syndrome unaffected by the endovascular treatment. In one patient (3%) prior treatment efforts in another hospital had caused ischemic stroke and two other patients (6%) initially presented with intracranial hemorrhage. In none of the cases were epileptic seizures, ischemia or hemorrhage was observed as a consequence related to the endovascular procedure at our institution (Tables 2 and 3).

#### Pulsatile tinnitus

14/30 patients (47%) who responded to our interview request suffered from pulsatile tinnitus upon admission, that in eight cases disappeared immediately and permanently following the intervention (57%). One patient (7%) reported transient relief but recurrence of the tinnitus during the LFU. One patient (7%) reported a secondary gradual recovery. In 4/14 patients (29%) the pulsatile tinnitus was unaffected by the endovascular treatment (Tables 2 and 3).

#### Overall statement on the long-term treatment result

The patient’s subjective benefit from treatment in the long-term follow-up was good. The vast majority of the patients (26/30, 87%) quoted satisfaction with the treatment result (“Better“). One patient (3%) reported no change (“Equal“). Three patients (10%) remained unsatisfied with the treatment result in the long-term comparison (“Worse“) (Tables 2 and 3).

## Discussion

The decision to treat an idCCF assumes that the potential benefit outweighs the risks of the intervention. Whereas indication is indisputable in fistulas associated with cortical venous drainage (CVD), neurologic deficits, intradural hemorrhage, venous thrombosis, or altered mental status [8] any other clinical presentation of an idCCF only offers a relative treatment indication. Some authors even recommend to not manage these lesions, expectantly awaiting potential spontaneous closure of the fistula [4,8,11,12].

Hence, in these cases, it is most important that treatment not only leads to good angiographic results, but also offers a perceived and sustainable benefit to the patient. Endovascular treatment of idCCF is preferentially performed in neurointerventional centers, however, little is known about the patient reported outcome several years after treatment and especially about how the patients themselves think about the therapy result.

In this article we aimed for a retrospective evaluation of the treatment results with a special focus on the patient’s view.

Our collective goes along with others published in literature in terms of sample size, baseline characteristics, primary treatment results and complication rate. One complication occurred among 33 treated patients (3%). In this specific case (pat. #21) endovascular treatment led to a secondary ophthalmic venous thrombosis resulting in a permanent ophthalmoplegia. This unpleasant fact matters even more because treatment in this case was initiated only due to a subjectively disturbing pulsatile tinnitus without eye-related symptoms or CVD.

This example, as well as the two unsuccessfully pre-treated patients show that catheter guided fistula embolization is not trivial and should be performed at a specialized facility by experts trained in this field. Even if therapy associated cranial nerve dysfunctions resolve in most of the cases [13], there remains a residual complication rate which underlines the importance of an indication evaluation process based on scientific evidence.

Any eye-related symptoms are successfully addressed by the endovascular treatment. If not ameliorated immediately this symptom group came up with a high percentage of secondary improvement and once achieved, the therapy success was sustainable in all of the cases. We strongly recommend treatment in any cases with altered intraocular pressure or progressive loss of visual acuity. Given the presence of appropriate expertise and facilities, treatment also should be offered to patients with any other eye-related symptoms like chemosis, exophthalmos or ophthalmoplegia, even if visual acuity or IOP is normal. Endovascular treatment is most likely to successfully and sustainably address the complaints in these constellations. We share this experience with other authors [8].

With an incidence of 42% upon initial presentation, pulsatile tinnitus is a common symptom of idCCF. Response to endovascular treatment was good. 57% of the patients initially presenting with this symptom were definitely and sustainably cured by the endovascular procedure. As in some cases a permanent tinnitus may significantly affect the patient’s quality of life, we conclude that endovascular treatment can be taken into account even if no other symptom is present.

3/8 patients (38%) progressed to complete fistula occlusion following subtotal endovascular treatment. Meyers et al. reported on a gradual decrease of fistula-related symptoms over a mean of 5.4 ± 1.3 months clinical follow-up after treatment. [8] This corresponds to our findings, hence waiting up to six months before planning another endovascular attempt is a reasonable option in a primary downgrading situation following subtotal endovascular fistula occlusion.

Endovascular treatment of CCFs has evolved over time. The optimal approach depends both on the arterial supply to the fistula and its venous drainage. Typically, transvenous approach for dural CCFs, is the procedure of choice when feasible and often favored due its high success rate, and lower risk of ischemic sequelae [8,14–16].

However, in some cases, an attempt at transarterial embolization may be performed first, especially if dural CCFs have accessible ECA supply [14,17,18].

In our collective a transarterial approach was chosen in 4/33 cases due to the individual vascular angioarchitecture and hemodynamics of the underlying fistula.

In particular patient # 13 received a partial transarterial gluebran embolization of the orbita draining fistula compartments of his small idCCF. In the case of patient # 14 a massive post-traumatic idCCF was occluded by a transarterial onyx embolization under temporary balloon protection of the ICA. Patient # 18 presented with a diffuse low-flow fistula network which was partially embolized using onyx via a microcatheter placed deep in an end branch of the external occipital artery. And patient # 19 finally was treated by a transarterial partial embolization of the fistula network using onyx over branches of the left ECA, whereas the neuromeningeal branch of the ascending pharyngeal artery was omitted due to the high risk of cranial nerve palsy.The vast majority of the patients (26/30, 87%) report good subjective satisfaction with the long-term treatment result, hence we state, that the patients themselves perceive their treatment to be highly beneficial to them. In addition to good rates of angiographic and clinical cure described in former publications [8,9], this is another important argument in favor of an endovascular treatment even if the indication constellation is only a relative one.

The subjective satisfaction with the treatment result cannot always be derived from the clinical symptoms and the angiographic result, as the examples of patients #14, #30 and #17 show:

Patient # 14 –as already mentioned—was admitted with a complete visual loss and fluctuating neurological deficits due to a massive post-traumatic idCCF with extensive AV-steal. Endovascular therapy completely occluded the fistula, led to a complete cessation of neurological failures and the patient regained his visual acuity. However an ophthalmoplegia persisted and the patient subsequently developed a chronic trophic corneal ulcer. Although he was objectively significantly less severely affected clinically after the intervention compared to before he stated to feel "worse" in the overall statement.

Patient # 30 was initially admitted with headache, chemosis, exophthalmos, unilateral abducens nerve palsy and papilledema. DSA showed an idCCF with major feeders from the right median meningeal artery and associated CVD. The fistula was completely occluded in one session by transvenous coiling of the cavernous sinus. The abducens nerve palsy and the diplopia disappeared immediately upon treatment. Whereas the imaging follow-up examinations showed no evidence of persistent or recurrent fistula, the patient reported headache, dizziness, tinnitus and a permanent reduction of visual acuity and quoted to feel „worse”in the LFU overall statement.

In contrast to these two patients the fistula of patient # 17 was successfully occluded in one uncomplicated treatment session which led to an immediate and complete relief of his initial symptoms (exophthalmos, chemosis, pulsatile tinnitus). In a follow-up consultation four years later, the patient reported recurrent headache behind the left ear, intermittent chemosis, pulsatile tinnitus and occasional diplopia. The patient underwent a control angiography (DSA) and several MRI checks including contrast-enhanced time-resolved MR angiography sequences which are known to reliably detect intracranial fistulas [19]. No recurrent fistula could be detected in any of the studies. Despite intermittent symptom recurrence the patient stated to feel “better” than before endovascular treatment in the overall statement in the LFU interview.

These examples show that the patient's subjective satisfaction with the therapy result and his perceived quality of life do not linearly dependent on angiographic outcome and clinical symptoms. Ultimately, satisfaction with the treatment result is a subjective statement that integrates not only objective factors, but also the patient’s individual perception, susceptibility to symptoms, and personal circumstances. To study this aspect of outcome was the aim of our study.”.

Some important limitations which hamper definitive conclusions of our study include the small sample size. Additionally clinical data was collected as it was documented in the medical records and does not result from a firmly designed prospective study protocol with standardized clinical examinations at pre-defined time points. For example, the imaging control examinations were not performed in a standardized manner at pre-defined time points which, in a way, affects the structural quality of our data. Also, a structured ophthalmologic examination by an ophthalmologist was documented in 16/33 (48%) cases prior to the intervention, whereas corresponding follow-up control examinations were performed in irregular manner in only a part of the patients. Just like in a work recently published by us about another patient collective with a similar methodology [10], this leaves an amount of uncertainty that a structured medical examination would have yielded a different result from the patient's subjective complaints. On the other hand, exactly the latter parameter was the main focus of our study, which is why we consider the resulting bias to be justifiable.

Summing up, endovascular treatment of idCCF has passed its final exam by not only offering excellent angiographic results and mostly immediate clinical cure, but also by proving its long-lasting stable subjective benefit even to our patients treated more than one decade ago.

Especially in constellations in which an idCCF offers only a relative indication (no CVD, neurologic deficits, intradural hemorrhage, venous thrombosis, or altered mental status), this is another important argument in favor of an endovascular treatment.

## Supporting information

S1 File Questionnaire. Long-term follow-up interview form provided to all patients.
(DOC)
